# Current status of preoperative risk assessment for posthepatectomy liver failure in patients with hepatocellular carcinoma

**DOI:** 10.1002/ags3.12692

**Published:** 2023-05-21

**Authors:** Takahiro Nishio, Kojiro Taura, Yukinori Koyama, Takamichi Ishii, Etsuro Hatano

**Affiliations:** ^1^ Department of Surgery, Graduate School of Medicine Kyoto University Kyoto Japan; ^2^ Department of Gastroenterological Surgery and Oncology Kitano Hospital Osaka Japan

**Keywords:** hepatocellular carcinoma, liver fibrosis, liver resection, portal hypertension, posthepatectomy liver failure

## Abstract

Liver resection is an effective therapeutic option for patients with hepatocellular carcinoma. However, posthepatectomy liver failure (PHLF) remains a major cause of hepatectomy‐related mortality, and the accurate prediction of PHLF based on preoperative assessment of liver functional reserve is a critical issue. The definition of PHLF proposed by the International Study Group for Liver Surgery has gained acceptance as a standard grading criterion. Liver function can be estimated using a variety of parameters, including routine blood biochemical examinations, clinical scoring systems, dynamic liver function tests, liver stiffness and fibrosis markers, and imaging studies. The Child–Pugh score and model for end‐stage liver disease scores are conventionally used for estimating liver decompensation, although the alternatively developed albumin‐bilirubin score shows superior performance for predicting hepatic dysfunction. Indocyanine green clearance, a dynamic liver function test mostly used in Japan and other Asian countries, serves as a quantitative estimation of liver function reserve and helps determine indications for surgical procedures according to the estimated risk of PHLF. In an attempt to improve predictive accuracy, specific evaluation of liver fibrosis and portal hypertension has gained popularity, including liver stiffness measurements using ultrasonography or magnetic resonance elastography, as well as noninvasive fibrosis markers. Imaging modalities, including Tc‐99m‐labeled galactosyl serum albumin scintigraphy and gadolinium‐enhanced magnetic resonance imaging, are used for preoperative evaluation in combination with liver volume. This review aims to provide an overview of the usefulness of current options for the preoperative assessment of liver function in predicting PHLF.

## Graphical abstract

Posthepatectomy liver failure remains a major cause of hepatectomy‐related mortality, and the accurate prediction of PHLF based on preoperative assessment of liver functional reserve is a critical issue. Multiple studies have evaluated the predictive performance of various preoperative parameters, which are broadly categorized as clinical scores based on routine blood tests, dynamic liver function tests, liver stiffness and noninvasive fibrosis markers, liver function imaging, and biomarkers for portal hypertension. This review aims to provide an overview of the utility of current options for preoperative assessment of liver function and the performance of each parameter in predicting PHLF.

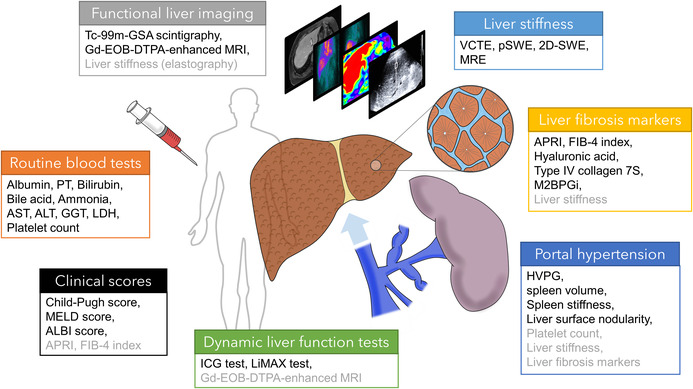

## 1 INTRODUCTION

Hepatocellular carcinoma (HCC) is the most common primary liver cancer and commonly arises in patients with chronic liver disease.^1^ There are many therapeutic options for HCC, including transplantation, ablation, transarterial chemoembolization, and systemic therapy. Liver resection is widely accepted as an effective curative treatment for HCC,^2^ and the capacity of the liver to regenerate and restore its function allows the removal of the part of the hepatic parenchyma with lesions. However, resection of an excessive volume of a diseased liver could result in insufficient functional reserve of the remnant liver, leading to posthepatectomy liver failure (PHLF).^3^ The mortality rate after liver resection in patients with HCC is reported to be up to 5.0%.^4^, ^5^, ^6^ Furthermore, PHLF significantly affects the long‐term survival of HCC patients.^7^, ^8^ Therefore, PHLF remains a major cause of hepatectomy‐related mortality and has been a barrier to expanding the surgical indications for HCC, despite advances in surgical techniques and perioperative management.^4^, ^9^, ^10^, ^11^, ^12^ Preventing the occurrence of PHLF by accurately estimating the liver functional reserve and determining the permissive future remnant volume during preoperative planning is essential.

Much effort has been made to develop selection criteria to identify patients at high risk of PHLF; however, accurate predictors of PHLF based on preoperative evaluation of liver function remain controversial. The aim of this narrative review is to provide an overview of the utility of current options for preoperative assessment of liver function and the performance of each parameter in predicting PHLF.

## 2 LITERATURE SEARCH

The predictors of PHLF are heterogeneous, and the criteria for surgical indications for patients with HCC differ greatly across institutions and countries. In addition, advances in surgical techniques and diagnostic tools, including imaging modalities and detection of novel biomarkers, have impacted perioperative management and postoperative outcomes in patients with HCC, further complicating the preoperative prediction of PHLF.

This article aims to review the current status and trends of preoperative risk assessment for liver resection and comprehend this heterogeneity. Because of a broad variety of liver function tests and the substantial heterogeneity of studies, a formal systematic review was not conducted. Rather, a pragmatic electronic literature search in the PubMed and Medline databases was performed using the keywords “liver failure OR liver insufficiency OR liver dysfunction OR liver decompensation” AND “posthepatectomy OR postoperative OR hepatectomy OR liver resection.” We particularly focused on literature published within the last 15 years. Only studies in humans published in English were considered. Studies were excluded if they fulfilled any of the following criteria: (1) a focus on liver resection for non‐HCC tumors, such as cholangiocarcinoma and metastatic cancer; (2) inclusion of only long‐term outcomes; (3) examination of correlation among the parameters or grading systems but not with outcomes; (4) lack of precise description of the definition of postoperative outcomes; and (5) no report on the detailed methodology.

## 3 DEFINITION OF PHLF

The incidence of PHLF has been reported to range from 1.2% to 32%, which potentially reflects the differences in patient demographics, pathology of underlying diseases, procedures performed, and the definition of PHLF.^13^ Various definitions of PHLF have been proposed, some of which have gained wide acceptance (Table 1).

**TABLE 1:** Representative definitions for PHLF.

Criterion	Study	Year	Definition	
50‐50 criteria	Balzan et al.^14^	2005	Prothrombin time index <50% and serum bilirubin >50 mmol/L (ie, 2.9 mg/dL) on postoperative day 5	
Peak bilirubin criterion	Mullen et al.^16^	2007	Postoperative peak bilirubin >7.0 mg/dL	
ISGLS	Rahbari et al.^13^	2011	An increased PT‐INR and concomitant hyperbilirubinemia on or after postoperative day 5	
			Grade A	PHLF resulting in abnormal laboratory parameters but requiring no change in the clinical management
			Grade B	PHLF resulting in a deviation from the regular clinical management but manageable without invasive treatment
			Grade C	PHLF resulting in a deviation from the regular clinical management and requiring invasive treatment

Abbreviations: ISGLS, International Study Group of Liver Surgery; PHLF, posthepatectomy liver failure; PT‐INR, prothrombin time‐international normalized ratio.

Balzan et al. in 2005 showed that a combination of prothrombin time <50% and bilirubin >50 μmol/L on postoperative day 5 was an accurate predictor of the risk of hepatectomy‐related mortality.^14^ These are termed the 50‐50 criteria, and their usefulness for the early diagnosis of PHLF has been validated.^15^ Mullen et al.^16^ proposed that a peak serum bilirubin concentration >7 mg/dL is a powerful predictor of 90‐day mortality and complications after major hepatectomy. Although the peak bilirubin criterion showed better predictive performance (area under the curve, 0.982; sensitivity, 93.3%; and specificity, 94.3%) than the 50‐50 criteria, the exclusion of patients with cirrhosis in this analysis has raised questions about its validity.

The International Study Group of Liver Surgery (ISGLS) proposed a standardized definition for PHLF in 2011.^13^ They defined PHLF as an increased prothrombin time‐international normalized ratio (PT‐INR) and concomitant hyperbilirubinemia on or after postoperative day 5. The severity is categorized into three grades, as follows: Grade A, resulting in abnormal laboratory parameters but requiring no deviation from standard care; Grade B, resulting in a deviation from regular clinical management and requiring noninvasive treatment; and Grade C, resulting in a deviation from the regular clinical management and requiring invasive treatment. The perioperative mortality rates of patients with grades A, B, and C PHLF are 0%, 12%, and 54%, respectively.^13^

The ISGLS definition has a higher sensitivity for predicting hepatectomy‐related mortality than the 50‐50 criteria and the peak bilirubin >7 mg/L criterion.^17^ Therefore, the ISGLS definition has gained acceptance as a standard grading criterion for PHLF and has been universally used in studies involving liver resection.^5^, ^18^, ^19^, ^20^, ^21^, ^22^

## 4 PREOPERATIVE ASSESSMENT FOR LIVER FUNCTIONAL RESERVE

**FIGURE 1:**
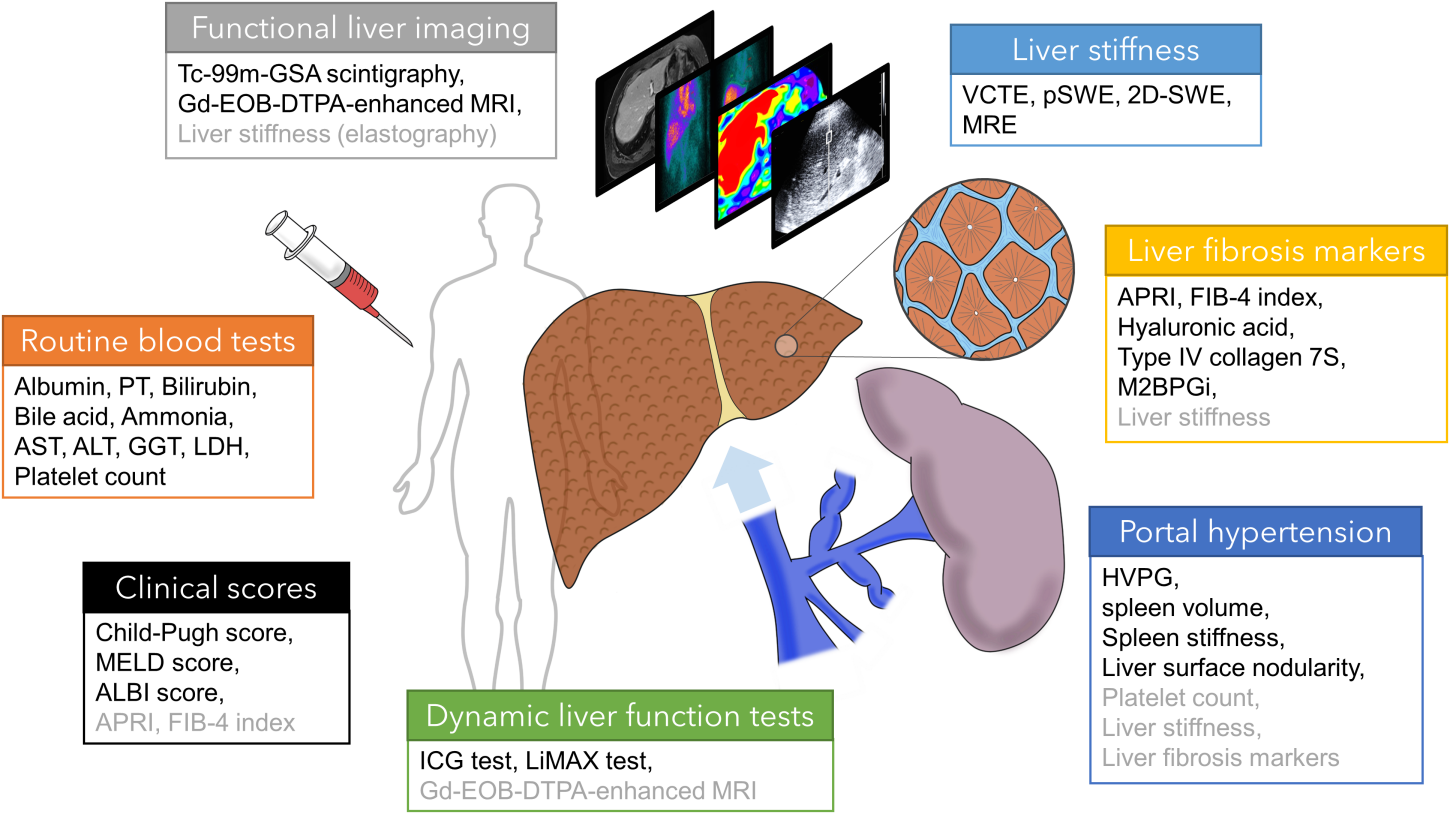
Schema of categories of liver function tests (related to Table 2). Preoperative tests to estimate liver functional reserve can be categorized into blood biochemical examinations, clinical scoring systems, dynamic liver function tests, fibrosis markers, including liver stiffness, imaging studies, and parameters of portal hypertension. Some items overlap across the categories, which are listed in gray text. 2D‐SWE, two‐dimensional shear wave elastography; ALBI, albumin‐bilirubin; ALT, alanine aminotransferase; APRI, aspartate aminotransferase to platelet ratio index; AST, aspartate aminotransferase; FIB‐4, fibrosis‐4; Gd‐EOB‐DTPA, gadolinium ethoxybenzyl diethylenetriamine pentaacetic acid; GGT, gamma‐glutamyl transferase; HVPG, hepatic venous pressure gradient; ICG, indocyanine green; LDH, lactate dehydrogenase; LiMAx, liver maximum capacity; M2BPGi, mac‐2 binding protein glycosylation isomer; MELD, model of end‐stage liver disease; MRE, magnetic resonance elastography; MRI, magnetic resonance imaging; pSWE, point shear wave elastography; PT, prothrombin time; Tc‐99m‐GSA, Technetium‐99m galactosyl serum albumin; VCTE, vibration‐controlled transient elastography.

The risk assessment for PHLF is mainly based on the optimization of preoperative liver function reserve.^3^ Liver function can be estimated using various preoperative parameters, including blood biochemical examinations, clinical scoring systems, dynamic liver function tests, fibrosis markers, including liver stiffness (LS), imaging studies, and parameters of portal hypertension, including the hepatic venous pressure gradient (HVPG)^5^, ^22^ (Table 2, Figure 1).

**TABLE 2:** Categories of liver function tests.

Liver function tests	Items/components
Routine blood tests	
(syntheticm excretory, detoxifying)	Albumin, PT, bilirubin, bile acid, ammonia
(liver enzymes)	AST, ALT, GGT, LDH
(portal hypertension)	Platelet count
Clinical scores	
Child–Pugh score	Serum bilirubin and albumin, PT, ascites, encephalopathy
MELD score	Serum bilirubin and creatinine, INR
ALBI score	Serum albumin and bilirubin
Dynamic liver function tests	
Indocyanine green test	ICG‐R15, KICG
LiMAx test	^13^CO_2_:^12^CO_2_ ratio in the expired breath
Serum liver fibrosis markers	
APRI	AST, platelet count
FIB‐4 index	Age, AST, ALT, platelet count
Hyaluronic acid	
Type IV collagen 7S	
M2BPGi	
Liver stiffness	
Ultrasound elastography	VCTE, pSWE, 2D‐SWE
Magnetic resonance elastography (MRE)	
Functional liver imaging	
Tc‐99m‐GSA scintigraphy	HH15, LHL15
Gd‐EOB‐DTPA‐enhanced MRI	RLE, HUI
Portal hypertension	
Hepatic venous pressure gradient	
Liver stiffness	
Spleen volume	
Spleen stiffness	
Liver surface nodularity	

Abbreviations: 2D‐SWE, two‐dimensional shear wave elastography; ALBI, albumin‐bilirubin; ALT, alanine aminotransferase; APRI, aspartate aminotransferase to platelet ratio index; AST, aspartate aminotransferase; FIB‐4, fibrosis‐4; Gd‐EOB‐DTPA, gadolinium ethoxybenzyl diethylenetriamine pentaacetic acid; GGT, gamma‐glutamyl transferase; HH15, blood clearance ratio of Tc‐99m GSA; ICG‐R15, indocyanine green retention rate at 15 minutes; INR, international normalized ratio; LDH, lactate dehydrogenase; LHL15, hepatic uptake ratio of Tc‐99m GSA; LiMAx, liver maximum capacity; M2BPGi, mac‐2 binding protein glycosylation isomer; MELD, model of end‐stage liver disease; MRI, magnetic resonance imaging; pSWE, point shear wave elastography; PT, prothrombin time; Tc‐99m‐GSA, Technetium‐99m galactosyl serum albumin; VCTE, vibration‐controlled transient elastography.

Blood biochemical tests, which are routinely performed prior to surgery, allow screening for general hepatic conditions, including bile synthesis and secretion (bilirubin), protein synthesis (albumin and prothrombin time), detoxification (ammonia), and hepatocyte damage (aspartate aminotransferase and alanine aminotransferase).^3^

The Child–Pugh and model for end‐stage liver disease (MELD) scores are widely used liver function scoring systems that are useful for prognostication of patients with HCC.^20^, ^23^, ^24^, ^25^, ^26^, ^27^ However, they may have limited accuracy for predicting PHLF.^28^, ^29^, ^30^ The albumin‐bilirubin (ALBI) score was developed based on the long‐term prognosis of patients with HCC.^31^ The usefulness of the ALBI score, its modifications for the assessment of postoperative liver dysfunction, and its superiority to the Child–Pugh and MELD scores have been described; however, its predictive accuracy for PHLF still needs to be investigated.^29^, ^30^, ^32^, ^33^, ^34^, ^35^, ^36^, ^37^, ^38^

The indocyanine green (ICG) clearance test enables quantitative estimation of liver function reserve. The ICG test has been mostly used in Japan and other Asian countries, where it has been effective in determining the indications for surgical procedures according to the estimated risk of PHLF.^39^, ^40^, ^41^, ^42^

More recently, specific evaluation of cirrhosis and portal hypertension have gained popularity as tools for liver functional assessment, including LS measurement (LSM) using ultrasonography or magnetic resonance elastography (MRE),^43^, ^44^, ^45^ noninvasive fibrosis markers such as the aspartate aminotransferase‐to‐platelet ratio index (APRI)^46^, ^47^ and the fibrosis‐4 (FIB‐4) index,^48^, ^49^ and the mac‐2 binding protein glycosylation isomer (M2BPGi).^50^, ^51^ These noninvasive biomarkers can potentially be used as substitutes for liver biopsy and HVPG, which are invasive methods with acknowledged limitations.^22^, ^52^, ^53^, ^54^, ^55^ Imaging modalities have also been proposed for the estimation of liver function. Tc‐99m‐labeled galactosyl serum albumin (Tc‐99m‐GSA) liver scintigraphy^56^, ^57^, ^58^ and gadolinium‐enhanced magnetic resonance imaging (MRI) using gadolinium ethoxybenzyl diethylenetriamine pentaacetic acid (Gd‐EOB‐DTPA)^59^, ^60^, ^61^ are used for preoperative evaluation in combination with liver volume. In addition, liver surface nodularity (LSN) has recently been reported to be an independent predictor of PHLF in patients with HCC,^62^, ^63^ although this novel method requires validation in further studies.

## 5 ROUTINE BLOOD BIOCHEMICAL TESTS AND CLINICAL SCORES

The Child–Pugh score is an essential tool for stratifying the prognosis of patients with HCC and as a general guide for indication for surgical resection^23^, ^24^ and is the gold standard grading system for liver function. This score is based on five simple parameters: encephalopathy, ascites, serum total bilirubin level, serum albumin level, and prothrombin time.^64^ The MELD score, which incorporates renal function and general liver functional indicators, was originally designed to predict survival in patients with cirrhosis after insertion of a transjugular intrahepatic portosystemic shunt^65^ and has been used to prioritize candidates for liver transplantation. The MELD score has also been applied for the early prediction of postoperative morbidity and mortality in patients undergoing liver resection,^27^ but its predictive performance in patients without advanced cirrhosis is controversial.^12^, ^66^

Although the Child–Pugh score has conventionally been used for risk assessment of surgical treatment for patients with HCC, it has limitations, such as subjective parameters and arbitrary cutoff points. The ALBI score has emerged as an evidence‐based scoring system to assess liver function in patients with HCC. This score includes only the albumin and bilirubin values and is therefore more objective.^31^ The ALBI score has been widely accepted as a prognostic tool and has a good correlation with survival, time to recurrence, and tolerability of surgical, locoregional, and systemic therapies for HCC.^67^, ^68^ In addition, the ALBI score is more capable of predicting postoperative outcomes and major complications, including PHLF, than the Child–Pugh and MELD scores.^29^, ^30^, ^33^, ^34^, ^35^, ^69^ However, the utility of the preoperative ALBI grade for estimating the risk for PHLF has been demonstrated mainly in retrospective studies, and further investigations with high‐quality designs to evaluate its predictive accuracy are needed.^38^

Although the ALBI score has gained popularity because of its simple and objective parameters for predicting prognosis in HCC patients, there have been attempts to determine more accurate predictive models for PHLF using routine blood tests, including prothrombin time, aminotransferase, and platelet count, as well as specific indicators, such as the ICG test, LSM, and imaging modalities.^6^ Importantly, the platelet count, which is a conventional item not included in the ALBI score, is known to have an impact on the postoperative outcome for HCC, as it reflects clinically significant portal hypertension (CSPH).^70^ In addition, the platelet count is an essential component of noninvasive diagnostic tools for liver fibrosis, including the APRI and FIB‐4 index. Multiple studies have identified the platelet count as a significant predictor of PHLF,^6^, ^71^ and models that incorporate the platelet count combined with albumin,^72^ the ALBI score,^73^ ICG test,^74^ and other predictive factors^5^, ^75^ have shown a better predictive performance than the ALBI score. Of note, these models include resection volume or future liver remnant (FLR) as a parameter, which allows surgeons to plan surgical procedures based on liver functional evaluation using simple and conventional factors. Recently, nomograms have been developed based on multiple independent preoperative predictors, which enable multidisciplinary risk assessment to determine the indications for hepatectomy in patients with HCC^20^, ^76^, ^77^ (Table 3).

**TABLE 3:** Predictors and prediction models for PHLF based on routine tests.

Study	Year	Country	Study type	No. of participants	Outcome	No. of outcome	Parameters	AUROC (95% confidence interval)
ALBI‐based								
Fagenson et al.^30^	2020	US	R	13 783	ISGLS Grade B,C	397 (2.9%)	ALBI	0.67
Shi et al.^34^	2021	China	R	767	50‐50	102 (12.3%)	ALBI, APRI, Cirrhosis, CSPH, tumor size	0.79 (0.74–0.84)
Takahashi et al.^35^	2022	Japan	R	361	ISGLS Grade B,C	39 (11%)	ALBI, Era of surgery, HCV, Fibrosis, Res	0.89 (0.83–0.96)
Platelet count‐based								
Prodeau et al.^5^	2019	France	P	343	ISGLS Grade B,C	132 (38%)	Plt, FLR, ITT‐laparoscopy	0.73
Lu et al.^73^	2019	China	R	2038	ISGLS Grade B,C	196 (9.6%)	Plt, Alb, Bil	0.69
Yamamoto et al.^72^	2020	Japan	R	876	ISGLS Grade B,C	92 (10.5%)	Plt, Alb, FLR	0.75 (0.70–0.80)
Mai et al.^75^	2020	China	R	353	ISGLS Grade B,C	87 (24.6%)	Plt, PT, Bil, AST, FLR	0.88 (0.84–0.93)
Multidisciplinary clinical assessment								
Berardi et al.^76^	2020	International	R	253	90‐day morbidity	108 (42.7%)	Comorbidity, CPS, Hb, Plt, Ascites, CSPH, Open/mini‐invasive, Major/minor resection	0.79 (0.73–0.84)
Dhir et al.^77^	2021	US	R	10 808	ISGLS Grade B,C	316 (2.9%)	Age, BMI, Sex, Diabetes, Dyspnea, Ascites, Steroid, Bleeding disorder, ASA, Biliary Stent, Neoadjuvant therapy, viral hepatitis, Concurrent partial resections, biliary reconstruction, hepatectomy procedure, Na, Alb, Bil, INR	0.78
Wang et al.^20^	2021	China	R	2661	ISGLS Grade B,C	254 (9.5%)	Bil, Alb, GGT, PT, CSPH, Major/minor resection	0.86 (0.80–0.91) external validation
ICG test								
Li et al.^84^	2015	China	R	235	50‐50	31 (13.2%)	ICG‐R15, Operative bleeding, sRem	0.88 (0.83–0.92)
Kim et al.^85^	2015	Korea	R	81	50‐50	9 (11.1%)	ICG‐R15, sFLR	0.99 w/ cirrhosis, 0.76 w/o cirrhosis
Wang et al.^28^	2018	China	R	185	ISGLS Grade B,C	23 (12.4%)	ICG‐R15	0.72 (0.65–0.79)
Maruyama et al.^82^	2018	Japan	R	20	ISGLS Grade B,C	4 (20%)	KICG, FLR	–
Russolillo et al.^87^	2019	Italy	R	400	ISGLS Grade B,C	34 (8.5%)	Alb, ICG‐R15	–
Honmyo et al.^74^	2021	Japan	R	335	ISGLS Grade B,C	54 (16.1%)	ICG‐R15, Plt, PT, Res, FLR	0.87 (0.78–0.92)

Abbreviations: Alb, albumin; ALBI, albumin‐bilirubin; APRI, aspartate aminotransferase to platelet ratio index; ASA, American Society of Anesthesiologists; AST, aspartate transaminase; AUROC, area under the receiver operating characteristic curve; Bil, bilirubin; CPS, Child–Pugh score; CSPH, clinically significant portal hypertension; FLR, future liver remnant; GGT, gamma‐glutamyl transferase; Hb, hemoglobin; HCV, hepatitis C virus; ICG, Indocyanine green; ICG‐R15, indocyanine green retention rate at 15 minutes; INR, international normalized ratio; ISGLS, International Study Group of Liver Surgery; ITT, intension to treat; KICG, plasma disappearance rate of indocyanine green; PHLF, posthepatectomy liver failure; Plt, platelet count; PT, prothrombin time; R, retrospective; Res, resection volume rate.

## 6 DYNAMIC LIVER FUNCTION TESTS

### 6.1 ICG test

The clearance of intravenously administered exogenous substances that are metabolized or excreted via liver perfusion has been used to quantitatively examine liver function.^40^ The ICG retention test is as a well‐accepted method for preoperatively assessing liver functional reserve and is routinely performed in Japan and other Asian countries.^41^ It is a dynamic method that measures the hepatic clearance of ICG 15 min after its intravenous injection (ICG‐R15). ICG clearance is usually delayed in patients with liver damage, and an increase in the ICG‐R15 reflects the degree of liver dysfunction. The Makuuchi criteria, a decisional algorithm for the extent of hepatectomy according to the ICG‐R15, have reduced hepatectomy‐related morbidity and mortality, especially during the developmental stage of liver surgery in Japan.^39^

The ICG test has gained popularity as a preoperative liver functional test, and it has superior predictive performance compared to the Child–Pugh and MELD scores.^28^ Although the ICG‐R15 is not a linear parameter, the plasma disappearance rate of ICG (KICG) is useful for the quantification of liver function when combined with the estimated‐FLR as the KICG of the remnant liver (remKICG),^78^ which is correlated with the occurrence of PHLF not only in HCC patients^79^, ^80^ but also in those with biliary cancer^81^ and individuals who have undergone portal vein embolization.^82^ Some studies have used predictive models incorporating the ICG‐R15,^74^, ^83^, ^84^, ^85^ including the Albumin‐Indocyanine Green Evaluation model,^86^, ^87^ which has better performance than the Child–Pugh score but is comparable with the ALBI grade for predicting PHLF (Table 3). The potential limitation of the ICG clearance test is that its result is affected by biliary obstruction and hemodynamic alterations, such as intrahepatic shunt, portal hypertension, and thrombosis.

### 6.2 Liver maximum capacity

The liver maximum capacity (LiMAx) test evaluates hepatic metabolism by measuring the ^13^CO_2_:^12^CO_2_ ratio in the exhaled breath, which is derived from the rate of metabolism of intravenously injected ^13^C‐methacetin. The LiMAx test result strongly correlates with liver function reserve, and a preoperative volume/function analysis combining FLR and LiMAx enables an accurate estimation of remnant liver function prior to surgery.^88^, ^89^ The LiMAx test has gained acceptance mostly in Western countries, and the LiMAx decision tree algorithm has improved preoperative assessments for PHLF and postoperative outcomes.^90^, ^91^

## 7 SERUM MARKERS FOR LIVER FIBROSIS

### 7.1 APRI and FIB‐4 index

Liver fibrosis is a common consequence of chronic liver injury, and the extent of fibrosis is highly correlated with liver functional reserve and prognosis in patients with HCC. Liver biopsy is the standard option for evaluating liver fibrosis, but it is an invasive procedure and has several limitations, such as complications, sampling errors, intra‐ and interobserver variability, and expense.^92^ To address these limitations, noninvasive liver fibrosis markers suitable for routine use have been developed. The APRI (aspartate transaminase/[upper limit of normal] × 100/platelet count [10^9^/L])^46^ and the FIB‐4 index ([age (in years) × aspartate aminotransferase (U/L)]/[platelet count (10^9^/L) × alanine aminotransferase (U/L)^1/2^])^48^ can be commonly assessed using simple and conventional parameters. These liver fibrosis indices show excellent accuracy in predicting significant fibrosis and cirrhosis, and they have recently gained attention as noninvasive tools for the diagnosis and prognostication of nonalcoholic steatohepatitis (NASH). This is important as the incidence of NASH is rapidly increasing worldwide, and it is becoming a major etiology of chronic liver disease.^93^, ^94^, ^95^, ^96^ The APRI and FIB‐4 index are also useful for estimating liver functional reserve, as they correlate with the risk of perioperative mortality^97^ and have better predictive accuracy for PHLF than the MELD and Child–Pugh scores^34^, ^98^, ^99^, ^100^, ^101^, ^102^ (Table 4).

**TABLE 4:** Predictors and prediction models for PHLF based on liver fibrosis markers.

Study	Year	Country	Study type	No. of participants	Outcome	No. of outcome	Parameters	AUROC (95% confidence interval)
Serum fibrosis marker								
Mai et al.^98^	2021	China	R	637	ISGLS Grade B,C	101 (15.9%)	APRI, FLR	0.82
Dong et al.^100^	2015	China	R	338	ISGLS Grade B,C	14 (4.1%)	FIB‐4, FLR	0.85 (0.76–0.94)
Feng et al.^101^	2019	China	R	205	ISGLS Grade B,C	24 (11.7%)	FIB‐4	0.74 (0.59–0.88)
Zhou et al.^102^	2020	China	R	495	ISGLS Grade A,B,C	46 (9.3%)	FIB‐4	0.74 (0.66–0.84)
Ueno et al.^104^	2009	Japan	R	52	Clinical, CD≥3	17 (32.7%)	HA, FLR	0.92 (0.84–1.00)
Yachida et al.^106^	2009	Japan	R	131	Clinical	27 (20.6%)	HA	0.80 (0.70–0.89)
Kubo et al.^108^	2004	Japan	R	251	Clinical	25 (10.0%)	Type IV collagen 7S	‐
Ishii et al.^109^	2020	Japan	R	215	ISGLS Grade A,B,C	18 (8.3%)	Type IV collagen 7S	‐
Okuda et al.^111^	2017	Japan	R	138	ISGLS Grade B,C	19 (13.8%)	Plt, M2BPGi, Res	0.81 (0.69–0.89)
Liver stiffness measurement								
VCTE								
Cescon et al.^128^	2012	Italy	P	92	Clinical	26 (28.9%)	VCTE	0.87 (0.78–0.93)
Wong et al.^129^	2013	China	P	105	Clinical	15 (14.3%)	VCTE	0.79 (0.65–0.93)
Rajakannu et al.^131^	2017	France	P	106	Clinical	9 (8.5%)	VCTE	0.81 (0.51–0.91)
Lei et al.^132^	2017	China	P	247	Clinical	37 (15.0%)	VCTE, INR	0.87 (0.80–0.91)
Chong et al.^133^	2017	China	P	255	ISGLS Grade B,C	46 (18%)	VCTE	0.65 (0.55–0.74)
Serenari et al.^134^	2020	Itaky, Korea	P	471	CCI≥26.2	50 (10.6%)	Alb, VCTE, Age, MELD	0.75 (0.72–0.78)
pSWE								
Harada et al.^136^	2012	Japan	P	50	Ascites CD≥3	10 (20%)	pSWE	0.90
Nishio et al.^130^	2016	Japan	P	177	ISGLS Grade B,C	21 (11.9%)	pSWE (ARFI), FLR	0.80 (0.70–0.87)
Han et al.^137^	2017	China	P	77	ISGLS Grade A,B,C	27 (35.1%)	pSWE	0.84
Hu et al.^138^	2018	China	P	216	ISGLS Grade A,B,C	64 (29.6%)	pSWE, Plt, Bil, CSPH, GGT	0.82 (0.73–0.92)
Shimada et al.^139^	2021	Japan	R	95	Ascites CD≥3	9 (9%)	pSWE, Res	0.71
Toriguchi et al.^140^	2022	Japan	P	267	Ascites CD≥3	35 (13.1%)	pSWE	0.79 (0.72–0.87)
2D‐SWE								
Lee et al.^141^	2021	Korea	P	125	CCI≥26.2	18 (14.4%)	2D‐SWE	0.85 (0.78–0.91)
Ju et al.^142^	2021	China	R	236	ISGLS Grade A,B,C	33 (14.0%)	2D‐SWE, Alb, HBsAg	0.79
Fu et al.^143^	2021	China	R	215	ISGLS Grade B,C	23 (10.7%)	2D‐SWE	0.80
Shi et al.^144^	2022	China	P	130	ISGLS Grade B,C	40 (30.8%)	2D‐SWE	0.72 (0.62–0.82)
Long et al.^145^	2022	China	P	119	ISGLS Grade B,C	38 (31.9%)	2D‐SWE	0.72 (0.61–0.82)
MRE								
Abe et al.^146^	2017	Japan	P	175	CD≥3	28 (16.0%)	MRE	0.81
Lee et al.^147^	2017	Korea	R	144	ISGLS Grade A,B,C	43 (29.9%)	MRE	0.74 (0.64–0.82)
Sato et al.^148^	2018	Japan	P	96	CD≥3	15 (16%)	MRE, Alb	0.84
Bae et al.^149^	2020	Korea	R	208	CCI≥26.2	28 (13.5%)	MRE, Open/mini‐invasive, Major/minor resection	0.91 (0.86 0.96)
Shibutani et al.^150^	2021	Japan	R	108	CD≥3	22 (20.4%)	MRE, FLR	0.82 (0.73–0.90)
Cho et al.^151^	2022	Korea	R	160	ISGLS Grade B,C	19 (11.9%)	MRE, ALBI, Alb, AFP, Major/minor resection	0.92 (0.87–0.97)

Abbreviations: 2D‐SWE, two‐dimensional shear wave elastography; Alb, albumin; APRI, aspartate aminotransferase to platelet ratio index; AUROC, area under the receiver operating characteristic curve; Bil, bilirubin; CCI, comprehensive complication index; CD, Clavien‐Dindo classification; FIB‐4, fibrosis‐4; FLR, future liver remnant; GGT, gamma‐glutamyl transferase; HA, hyaluronic acid; HBsAg, hepatitis B surface antigen; INR, international normalized ratio; ISGLS, International Study Group of Liver Surgery; M2BPGi, Mac‐2 binding protein glycosylation isomer; MELD, model of end‐stage liver disease; MRE, magnetic resonance elastography; P, prospective; PHLF, posthepatectomy liver failure; Plt, platelet count; pSWE, point shear wave elastography; R, retrospective; Res, resection volume; Res, resection volume rate; VCTE, vibration‐controlled transient elastography.

### 7.2 Specific liver fibrosis markers

Other specific markers for liver fibrosis examined by blood tests include hyaluronic acid, type IV collagen 7S, and M2BPGi. Serum hyaluronic acid, which reflects sinusoidal endothelial cell function correlated with hepatic fibrosis, is a reliable indicator of liver functional reserve and a useful predictor of postoperative complications.^103^, ^104^, ^105^, ^106^, ^107^ Type IV collagen 7S is a biomarker of liver fibrogenesis, and its serum concentration correlates with hepatic dysfunction following liver resection.^108^, ^109^ More recently, M2BPGi, which is a unique fibrosis‐related glyco‐alteration detected by a glycan sugar chain‐based immunoassay, has been proposed as a novel marker for liver fibrosis.^110^ Serum M2BPGi levels have a predictive accuracy for the diagnosis of liver fibrosis progression comparable to that of LSM and superior to that of APRI, hyaluronic acid, and type IV collagen 7S.^50^, ^51^ Further, M2BPGi can predict PHLF better than other preoperative parameters, including KICG, especially in patients with HCV‐related HCC.^111^ Taken together, the results of these studies indicate that serum markers for liver fibrosis are useful for the preoperative assessment of liver functional reserve. However, these results are mostly based on retrospective analysis in a limited number of centers, and further well‐designed prospective studies are required to determine the markers with the best accuracy.

## 8 LSM

Liver stiffness measurement has been widely accepted as a noninvasive assessment procedure for liver fibrosis and is an alternative to liver biopsy. The high diagnostic accuracy of LSM is based on the pathogenesis of liver fibrosis, in which the deposition of excessive amounts of extracellular matrix due to chronic injury increases tissue elasticity, enabling quantification of the extent of liver damage. LS is also affected by inflammation, passive venous congestion, portal hypertension, and biliary obstruction, which are potential confounders. Recently, LSM has gained popularity as a noninvasive assessment of liver fibrosis in patients with NAFLD and NASH because of the rapid increase in NAFLD/NASH‐related end‐stage liver diseases worldwide.^112^, ^113^, ^114^

### 8.1 Ultrasonographic elastography

Liver stiffness measurement is performed using ultrasonography‐ or MRI‐based techniques. Ultrasonographic elastography includes vibration‐controlled transient elastography (VCTE), point shear wave elastography (pSWE), acoustic radiation force impulse (ARFI), and two‐dimensional shear wave elastography (2D‐SWE).^45^, ^115^ VCTE has been validated in a large number of studies with good reproducibility, but it has the limitation of a lack of imaging. pSWE and 2D‐SWE can be performed in combination with real‐time standard B‐mode imaging in which the region of interest can be adjusted by the operator; the former technique acquires point measurements, and the latter yields a 2D elastographic tissue map. There is good to excellent agreement across different ultrasonographic systems for LSM.^116^, ^117^ The diagnostic performances of each ultrasonographic technique have been described in several meta‐analyses, showing good to excellent accuracy for diagnosing liver fibrosis stage.^118^, ^119^, ^120^ The potential limitations of ultrasonographic elastography include the need for training, limited availability, high cost, and failure due to artifacts, operator inexperience, ascites, obesity, narrow intercostal space, and confounders, such as inflammation, venous congestion, cholestasis, non‐fasting, and exercise.^121^

### 8.2 MRE

Magnetic resonance elastography was developed as a noninvasive imaging method for quantifying liver fibrosis with high accuracy.^122^ It can be easily incorporated into current abdominal MRI protocols and is capable of providing a stiffness map of the entire liver as well as a comprehensive evaluation in conjunction with MRI across the abdomen. MRE is reliable and repeatable with high intra‐ and interobserver agreement and without significant variability across vendors.^123^, ^124^, ^125^, ^126^ However, there are limitations, including cost, availability, and patient‐dependent factors such as the presence of magnetically susceptible implants, compliance with breath‐hold, and claustrophobia.^121^

### 8.3 Usefulness of LSM for prediction of PHLF

Liver stiffness measurement techniques have the potential to be applied to risk assessment for PHLF based on the significant correlation between the progression of liver fibrosis and extent of liver dysfunction.^127^ The utility of VCTE for predicting postoperative complications has been described.^128^, ^129^ A prospective study demonstrated that ARFI‐based LSM is useful for predicting PHLF based on the ISGLS definition, with higher accuracy than conventional preoperative tests, including KICG, and other fibrosis markers, such as hyaluronic acid, type IV collagen, the APRI, and the FIB‐4 index.^130^ The PHLF prediction model of the ARFI value incorporating FLR allows surgeons to make decisions regarding surgical procedures based on the estimation of permissive resection volume and has superior predictive performance to remKICG. Similarly, several studies have validated the usefulness of ultrasonographic elastography as a preoperative assessment modality for predicting postoperative complications across ultrasonographic systems, including VTCE,^131^, ^132^, ^133^, ^134^, ^135^ pSWE,^136^, ^137^, ^138^, ^139^, ^140^ and 2D‐SWE.^141^, ^142^, ^143^, ^144^, ^145^ LSM by MRE can also be used as a risk assessment modality for major complications after liver resection, including PHLF, and has good predictive performance compared to the ICG test, MELD score, APRI, FIB‐4 index, and VCTE value^146^, ^147^, ^148^, ^149^, ^150^, ^151^ (Table 5). LSM is a promising technique for evaluating preoperative liver functional reserve, and further investigations are required to develop LSM‐based criteria for determining surgical indications.

**TABLE 5:** Predictors and prediction models for PHLF based on imaging and CSPH parameters.

Study	Year	Country	Study type	No. of participants	Outcome	No. of outcome	Parameters	AUROC (95% confidence interval)
Liver functional imaging								
Tc‐99m‐GSA								
Kaibori et al.^156^	2008	Japan	R	191	Clinical	16 (8.3%)	GSA‐Rmax, HA	–
Hayashi et al.^159^	2015	Japan	R	133	CD≥3	10 (7.5%)	LHL15, FLR	–
Nakamura et al.^161^	2018	Japan	R	218	ISGLS Grade B,C	38 (17.4%)	LHL15, FLR	–
Kato et al.^56^	2018	Japan	R	100	ISGLS Grade A,B,C	33 (33%)	LHL15, FLR; Rmax, FLR	0.79 (0.67–0.87) LHL15‐FLR 0.78 (0.67–0.86) Rmax‐FLR
Okabayashi et al.^163^	2017	Japan	P	185	ISGLS Grade A,B,C	14 (8%)	LHL15, FLR	–
Tomita et al.^165^	2021	Japan	R	102	ISGLS Grade B,C	15 (14.7%)	LU15, FLR	0.82 (0.70–0.93)
EOB‐MRI								
Araki et al.^61^	2020	Japan	R	129	ISGLS Grade B,C	5 (3.9%)	LMR, FLR	0.94 (0.89–0.99)
Orimo et al.^172^	2020	Japan	R	140	ISGLS Grade B,C	13 (9.3%)	HUI, FLR	0.87
Tsujita et al.^173^	2020	Japan	R	41	ISGLS Grade B,C	9 (21.9%)	HUI, FLR	0.89 (0.73–0.96)
Wang et al.^174^	2021	China	R	116	ISGLS Grade A,B,C	28 (24.1)	RE, FLR	0.88 (0.81–0.93)
Luo et al.^175^	2022	China	R	502	ISGLS Grade A,B,C	90 (17.9%)	FLIS	0.75 (0.71–0.79)
CSPH parameter								
Bae et al.^183^	2021	Korea	R	317	ISGLS Grade A,B,C	72 (22.7%)	SV	0.66 (0.61–0.71)
Fernández‐Placencia et al.^184^	2020	France	R	107	Clinical PHD	9 (8.8%)	SV, LS	0.96 (0.93–0.98)
Peng et al.^185^	2019	China	P	158	ISGLS Grade A,B,C	23 (14.6%)	SV, FLR; SS	0.86 (0.76–0.95) SV/FLR 0.87 (0.79–0.94) SS
Hobeika et al.^62^	2020	France	R	187	ISGLS Grade B,C	12 (6.4%)	LSN	0.72

Abbreviations: AUROC, area under the receiver operating characteristic curve; CSPH, clinically significant portal hypertension; EOB‐MRI, gadolinium ethoxybenzyl diethylenetriamine pentaacetic acid‐enhanced magnetic resonance imaging; FLIS, functional liver imaging score; FLR, future liver remnant; HUI, hepatocellular uptake index; ISGLS, International Study Group of Liver Surgery; LHL15, hepatic uptake ratio of Tc‐99m‐GSA; LMR, liver to muscle ratio; LS, liver stiffness; LSN, liver surface nodularity; LU15, liver uptake Tc‐99m‐GSA; P, prospective; PHLF, posthepatectomy liver failure; R, retrospective; RE, relative enhancement; Rmax, maximal removal rate of Tc‐99m‐GSA; SS, spleen stiffness; SV, spleen volume; Tc‐99m‐GSA, Technetium‐99m galactosyl serum albumin scintigraphy.

## 9 FUNCTIONAL LIVER IMAGING

### 9.1 Hepatobiliary scintigraphy

Tc‐99m‐GSA scintigraphy is a well‐accepted imaging modality for assessing liver function.^152^ Tc‐99m‐GSA is a liver scintigraphy agent that binds to the asialoglycoprotein receptor on hepatocytes.^153^ Hepatic dysfunction as detected by the abnormal distribution of functioning hepatocytes with Tc‐99m‐GSA is correlated with hepatic disorders, including steatosis, fibrosis, and necrosis due to chronic liver injury. The HH15 (blood clearance ratio), representing retention of the tracer in the blood, and LHL15 (hepatic uptake ratio), representing uptake of the tracer in the liver, are commonly used as parameters. One of the benefits of Tc‐99m‐GSA scintigraphy is its ability to quantify the function of specific parts of the liver as well as the function of the entire liver,^154^ which is useful for estimating the function of the FLR during preoperative risk assessment and decision‐making in surgical procedures. Tc‐99m‐GSA is also applicable for patients undergoing portal vein embolization or two‐stage procedures, as liver function in such patients is not uniform across liver segments.^155^ Tc‐99m‐GSA scintigraphy‐derived parameters show good correlation with postoperative outcomes, including PHLF.^56^, ^83^, ^156^, ^157^, ^158^, ^159^, ^160^, ^161^ The fusion images of SPECT and computed tomography (CT) scans can provide a simultaneous assessment of anatomical details and the corresponding functions.^58^, ^162^, ^163^, ^164^, ^165^ Tc‐99m‐mebrofenin scintigraphy is also used to obtain functional liver imaging to estimate FLR function and the risk of PHLF after major hepatectomy and two‐stage procedures.^83^, ^166^, ^167^ Tc‐99m‐mebrofenin scintigraphy correlates with ICG retention because its absorption, excretion, and lack of hepatic biotransformation are similar to those of ICG.^168^

### 9.2 Gd‐EOB‐DTPA‐enhanced MRI

Gd‐EOB‐DTPA‐enhanced MRI (EOB‐MRI) has been proposed for the evaluation of liver functional reserve, as the specific uptake of Gd‐EOB‐DTPA by hepatocytes reflects their function. Signal intensity‐based parameters, including the relative liver enhancement, hepatic uptake index,^169^ and liver imaging score,^170^ are commonly used to evaluate liver function, and these EOB‐MRI‐derived parameters are effective for the preoperative prediction of PHLF.^61^, ^171^, ^172^, ^173^, ^174^, ^175^ The advantages of EOB‐MRI include high spatial resolution and combined anatomical and functional assessment, which enable the evaluation of regional liver function and diagnosis of hepatic lesions prior to liver resection. Moreover, it is reasonable to incorporate the FLR volume for precise estimation of postoperative residual function.^61^, ^172^, ^173^, ^174^ EOB‐MRI is better at evaluating the regional liver function reserve than other modalities, such as Tc‐99m‐GSA scintigraphy, because of its superior spatial resolution.^176^ EOB‐MRI‐based parameters have good to excellent accuracy (AUC 0.75–0.96) in predicting PHLF, and they have better accuracy than the ICG test, MELD score, and ALBI score.^173^, ^174^, ^175^ However, the heterogeneity in the variance of EOB‐MRI‐derived parameters and limited sample size in the existing studies highlight the necessity for well‐designed, prospective, multicenter studies with large sample sizes. Further, liver functional imaging, including hepatobiliary scintigraphy and MRI, are more costly than non‐imaging tests. Further investigations are necessary to determine whether the potential benefits of these imaging tests can overcome this disadvantage because of the overall cost reduction due to the improvement of patient outcomes.

## 10 ASSESSMENT FOR PORTAL HYPERTENSION

### 10.1 HVPG

Clinically significant portal hypertension is strongly correlated with hepatic decompensation and mortality in HCC patients. CSPH is not necessarily a contraindication for liver resection^10^ because minor resection in patients with moderate CSPH yields competitive survival outcomes.^23^ The extent of hepatectomy should be determined based on preoperative risk assessment of the severity of portal hypertension as well as other liver functional indicators to prevent the occurrence of PHLF, especially in patients with cirrhosis. HPVG is the gold‐standard direct assessment of portal hypertension and a significant predictor of hepatic decompensation and patient survival. Preoperative HVPG is associated with postoperative liver dysfunction and mortality after liver resection in patients with HCC and liver cirrhosis.^53^ CSPH is defined as an HVPG >10 mg, and its relevance to a higher risk of PHLF has been proposed.^177^ Nevertheless, HVPG is not routinely measured in clinical practice, as it is a potentially invasive technique with complex procedures and limited reproducibility due to inter‐operator variability.

### 10.2 Noninvasive assessment for CSPH

Alternative noninvasive parameters have been used to assess CSPH. The standard surrogate criteria include the presence of gastroesophageal varices or thrombocytopenia (platelet count <100 000/mL) and splenomegaly (diameter >12 cm).^70^ LS can also estimate portal hypertension, allowing highly accurate noninvasive identification of CSPH,^54^ particularly when combined with spleen diameter and platelet count.^178^ Spleen stiffness measured by elastography, as well as spleen volume,^179^, ^180^, ^181^, ^182^ better reflect CSPH, and these splenomegaly‐related parameters are useful for predicting PHLF.^183^, ^184^, ^185^ More recently, CT‐based LSN has been proposed as a diagnostic tool for detecting CSPH.^186^ The LSN score is associated with severe complications and PHLF after liver resection.^22^, ^62^ Quantification of LSN can be performed using routine CT images, and it may be a promising method for assessing liver functional reserve in the preoperative setting; however, further large cohort studies are needed to confirm its accuracy.

## 11 CONCLUSION

Accurate prediction of PHLF baswed on preoperative assessment of liver functional reserve remains challenging, and much effort has been made to develop criteria to ensure the safety of liver resection in patients with HCC. Multiple studies have evaluated the predictive performance of various preoperative parameters, which are broadly categorized as clinical scores based on routine blood tests, dynamic liver function tests, LS and noninvasive fibrosis markers, liver function imaging, and biomarkers for CSPH. These categories are not completely independent, and some parameters overlap across groups. For example, platelet count, generally included in routine blood tests, is a composition of fibrosis markers, including the APRI and FIB‐4 index, and it is also useful for estimating the presence of CSPH, and LSM, which is mostly based on imaging analysis, accurately evaluates CSPH and liver fibrosis. Additionally, imaging modalities, including EOB‐MRI and hepatobiliary scintigraphy, are also categorized as dynamic liver function tests. Of note, imaging techniques can simultaneously evaluate function and anatomy and preoperatively provide useful information for estimating safe and feasible FLR.

Although the superiority of single predictors is controversial, a combination of parameters with consideration of their role in each category should enable comprehensive risk assessment for PHLF, leading to the proposal of predictive models based on the clinical background of individual patients. This review helps organize the current status of the preoperative prediction of PHLF, highlighting the necessity for further well‐designed, large investigations to identify the best combination of parameters for the establishment of novel criteria for liver resection.

## AUTHOR CONTRIBUTIONS

TN and KT conceived the idea of the study. TN, KT, YK, and TI significantly contributed to literature search and review. TN and KT substantially contributed to the manuscript drafting. EH supervised the conduct of this study. All authors critically reviewed and revised the manuscript draft and approved the final version for submission.

## FUNDING INFORMATION

Supported by the Grant‐in‐Aid for Scientific Research from the Japan Society for the Promotion of Science, *KAKENHI No. 21K16444* (TN) and *22K08733* (YK); and by the Takeda Science Foundation (TN).

## CONFLICT OF INTEREST STATEMENT

The authors declare no conflicts of interest for this article.

## ETHICS STATEMENTS

Approval of the research protocol: N/A.

Informed Consent: N/A.

Registry and the Registration No. of the study/trial: N/A.

Animal Studies: N/A.
